# HIV and its associated factors among people who inject drugs in Mizoram, Northeast India

**DOI:** 10.1371/journal.pone.0286009

**Published:** 2023-05-22

**Authors:** Lucy Ngaihbanglovi Pachuau, Caterina Tannous, Richard Lalramhluna Chawngthu, Kingsley Emwinyore Agho

**Affiliations:** 1 School of Health Sciences, Western Sydney University, Campbelltown, NSW, Australia; 2 Mizoram State Aids Control Society, Aizawl, Mizoram, India; 3 Translational Health Research Institute (THRI), Western Sydney University, Campbelltown, Australia; 4 African Vision Research Institute (AVRI), University of KwaZulu-Natal, Westville Campus, Durban, South Africa; Guangxi Medical University, CHINA

## Abstract

**Aim:**

To estimate the prevalence and associated factors with the human immune-deficiency virus (HIV) among people who inject drugs (PWID) in Mizoram, Northeast India.

**Methods:**

The data source for the analysis was the 2019–2020 Mizoram State AIDS Control Society (MSACS) survey from 2695 PWID registered for the Targeted Intervention (TI) services. Logistic regression analysis was conducted to examine the factors associated with HIV among PWID after adjusting for sociodemographic characteristics, injection, and sexual behaviours.

**Results:**

21.19% of the participants tested positive for HIV and the prevalence of HIV among male and female participants were 19.5% and 38.6%, respectively. Multiple logistic regression analysis revealed that female (AOR 1.74; 95% CI 1.26–2.41), 35 years or older (AOR 1.45; 95% CI 1.06–1.99), married (AOR 1.41; 95% CI1.08–1.83), divorced/separated/widowed (AOR 2.12; 95% CI 1.59–2.82) and sharing of needle/syringe (AOR 1.62; 95% CI 1.30–2.00) were all positively associated with HIV infection. We also found that concomitant alcohol use was reduced by 35% (AOR 0.65; 95% CI 0.51–0.82) among HIV positive PWID, and HIV infection was also reduced by 46% (AOR 0.54; 95% CI 0.44–0.67) among those PWID who use a condom with a regular partner.

**Conclusion:**

The findings of this study suggested that there is a high prevalence of HIV among PWID with 1 in 5 PWID reported to have HIV. HIV among PWID was significantly higher among those over 35 years of age, females and divorced/separated/widowed participants. Needle/syringe sharing behaviour is an important determinant of HIV infection. The high prevalence of HIV among PWID population is multifactorial. To reduce HIV among PWID in Mizoram, interventions should target those sharing needles/syringes, females, especially those over 35 years of age and unmarried participants.

## 1. Introduction

Injecting drug use is a significant public health concern leading to the risk of blood-borne infections mainly Human Immunodeficiency Virus (HIV) [1]. An estimated 11.3 million people worldwide inject drugs [1]. Injecting drug use is responsible for an increasing proportion of new HIV infections in many parts of the world [2]. The risk of HIV for people who inject drugs (PWID) is 22 times higher than for people who do not inject drugs [3]. Outside of Sub-Saharan Africa, 25 percent of new HIV infections are among people who inject drugs and their sexual partners [3]. Eastern Europe and Central Asia saw a 29 percent increase in new HIV infections between 2010 and 2017, this burden is particularly high among PWID [4].

There are an estimated 200,000 PWID in India and HIV prevalence among them is estimated to be 6.23% [5, 6]. The current report on the overview of the HIV epidemic in India shows that the adult prevalence of HIV is highest among PWID [7]. In India, Targeted Intervention (TI), under the National AIDS Control Program (NACP) is the most effective means of controlling the spread of HIV amongst persons most vulnerable to HIV/AIDS, including PWID [8]. Targeted Interventions (TI’s) include the provision of needle and syringe exchange programs, condom distribution, opioid substitution therapy, treatment of sexually transmitted infections and behaviour change communication [9–11].

Injection drug use has been a major driver of the HIV epidemic in Northeastern states of India, given their proximity to the golden triangle of heroin production (Myanmar, Laos, Thailand) [12, 13]. In Mizoram, one of the northeastern states of India, an estimated 31.8 percent of HIV infections occur in injecting drug users [14]. PWID are at risk of HIV transmission which arises from sharing needles and injection equipment, injecting drugs and sex work, criminalization, marginalization and poverty [4, 15]. People who inject drugs are often subjected to marginalization and stigmatization by their family and community, which creates social and economic barriers to accessing public health interventions [16]. Low HIV testing uptake and lack of knowledge of the risks associated with injecting drug use may contribute to the high rates of HIV infection [17].

Mizoram has had a long- standing battle with injecting drug abuse among its youth since the entry of heroin in to the state in the 1980s [18]. According to the 2017 HIV Sentinel Surveillance report, the highest HIV prevalence among PWID in India was recorded in Mizoram (19.8%) [19]. The most recent study of HIV prevalence among PWID in 15 Indian cities reported that in Aizawl, the capital city of Mizoram, 72.8 percent of PWID reported sharing needles, and 57.9 percent reported having unprotected heterosexual sex in the past six months [20]. The study also showed that females had a greater than 3 fold higher odds of HIV than male PWID which may be mediated by dual injection-related and sexual risks which is similar to the findings of Mahanta and co-workers [21] who also found a significant association for HIV infection in females compared to males.

HIV prevention efforts in the Northeast of India have been underway since the mid-1990s through targeted interventions (TI’s). In Mizoram, the Mizoram State AIDS Control Society (MSACS) employs TI’s among high-risk groups, but there is not enough evidence or study to show that these strategies have been effective in curbing HIV among PWID within the state of Mizoram. Social Network Model (SNM) has been introduced in the capital city, Aizawl, aiming to cover injecting drug users who have never registered at TI and have never received any HIV related services [22].

Despite these efforts, Mizoram continues to have the highest HIV prevalence and HIV incidence in the country [23] and injecting drug use has contributed to the increasing new HIV infections in the state [24]. To better understand the extent of new infections and factors contributing to the increase, this study aims to estimate the prevalence of HIV among PWID and identify the risk factors of HIV among HIV positive PWID in Mizoram. The findings from this study will produce a greater understanding of the factors that influence HIV infection in this population. The recommendations will guide policymakers in making data informed decisions regarding the ways in which programs can be adjusted to support HIV prevention among this key population in Mizoram. The study findings will also guide further research on HIV/AIDS among this population group.

## 2. Methods

### 2.1. Study design and setting

The study used secondary data from the Mizoram State AIDS Control Society (MSACS). Mizoram lies in the North-East of India, nestled between two international borders with Bangladesh and Myanmar. The most recent data collected by MSACS between January 2019 and December 2020 were used for this study. MSACS is an organization created by the Government of Mizoram on behalf of the state to respond to the HIV/AIDS epidemic and to deliver effective and efficient implementation of the AIDS control program.

The study participants were PWID recruited from 8 districts across Mizoram. Participants were recruited by peer educators (PE) and targeted outreach by outreach workers (ORW). Mapping exercises were done to identify hotspot areas where PWIDs congregated in different districts of Mizoram. For recruitment, ORWs and PEs visited hotspot areas to invite PWIDs to enrol in TI services. PWIDs were not recruited at the first encounter, ORWs and PEs were encouraged to build rapport and trusting relationship with PWIDs to minimize dropouts from the services. After several encounters, PEs and ORWs invited PWIDs to enrol in the TI services. Once consent was given each newly recruited PWIDs were given a Unique Personal Identification Number and were registered and enrolled in TI programme [25].

Data were collected mainly through Non-Government Organizations that supported Targeted Intervention (TI-NGO) programmes. There are 34 TI-NGOs in eight (8) districts in Mizoram. These TI-NGOs work in partnership with the National AIDS Control Organization (NACO) and the State AIDS Control Organization. Targeted Interventions (TI) are preventive interventions focused on High-Risk Groups (HRG). The TI projects are peer-led interventions implemented through NGOs/community based organizations (CBO) [25]. A comprehensive detailed description of the methodology, sampling procedure, recruitment procedures and biological data collection procedure used is described elsewhere [26, 27].

### 2.2. Ethical considerations

Written ethics clearance and approval (No.D.12019/1/2020-MSACS (RA) were given by Mizoram State AIDS Control Society (MSACS) on 2^nd^ February 2021 for this study.

### 2.3. Inclusion and exclusion criteria

Individuals would be included in the study if they: (i) were 18 years or older; (ii) injected any illicit drugs (iii) resided only in Mizoram.

## 3. Outcome and confounding factors

One of the most common causes of HIV infection in India is injecting drug use [28]. Hence, the outcome of interest in this study was HIV infection among PWID and was coded as binary, 1 for ‘yes’ and 0 for ‘No’. The potential confounding factors included in this study were guided and consistent with previous studies [29–31] and were classified into three main factors namely, Socio-demographic factors, Substance use and injecting behaviour factors and Sexual behaviour factors. The Sociodemographic characteristics included age in category (‘18–24’, ‘25–34’ & 35+), gender (male/female), marital status (never married, married, separated/divorced/widowed), educational status (primary, middle, higher, graduate and above), employment status (unemployed, employed, self-employed), and average monthly income in Indian rupees (INR) (None, <3000, 3001–6000, 6001–10,000, >10,000). Substance use and injecting behaviour factors which relate directly to injecting any drugs in the past 3 months (Yes/No)., type of drugs used (heroin, parvon), sharing of needle/syringe (Yes/No) and concomitant alcohol use (Yes/No). Factors related to sexual behaviour were whether the person was engaged in sex work (Yes/No), used a condom with a regular partner (Yes/No) and used a condom with a non-regular/paid partner (Yes/No).

### 3.1. Statistical analysis

Exploratory data analysis on sociodemographic characteristics, substance use and injecting behaviour and sexual behaviour factors was conducted using frequency distribution. The study characteristics were described using frequency tabulation. A Chi-square test was used to compare the differences between HIV among people who inject drugs by sociodemographic characteristics, substance use and injecting behaviour and sexual behaviour factors.

Bivariate analyses were performed to examine the independent association between outcome (HIV among people who inject drugs) and all potential confounding variables (socio-demographic characteristics, substance use and injecting behaviour and sexual behaviour factors) and those potential confounding variables with *p*<0.20 were retained and used in the multiple logistic analyses [32]. Multiple logistic analyses using manual process backward elimination process were used for identifying factors associated with HIV among people who inject drugs in adults aged 18 years and over. The adjusted prevalence and their corresponding 95%CI were obtained using the “margins, atmean” command in STATA. There is no specific statistical test for the multicollinearity of the independent variables for binary logistic regression, but in this study, binary outcome variables were treated as a continuous variable by using the “Logit” command and then ‘collin’ command was used to examine multicollinearity including Variance Inflation Factors (VIF) and VIF<4 was considered suitable [33] and the model fitness was examined using the Hosmer-Lemeshow statistic [34]. We considered *p*≤0.05 as statistically significant. All statistical tests were two-tailed, and analyses were undertaken with the Stata software (version 17) (2021; Stata Corporation, College Station, TX, USA).

## 4. Results

### 4.1. Prevalence of HIV infection

**Fig 1:**
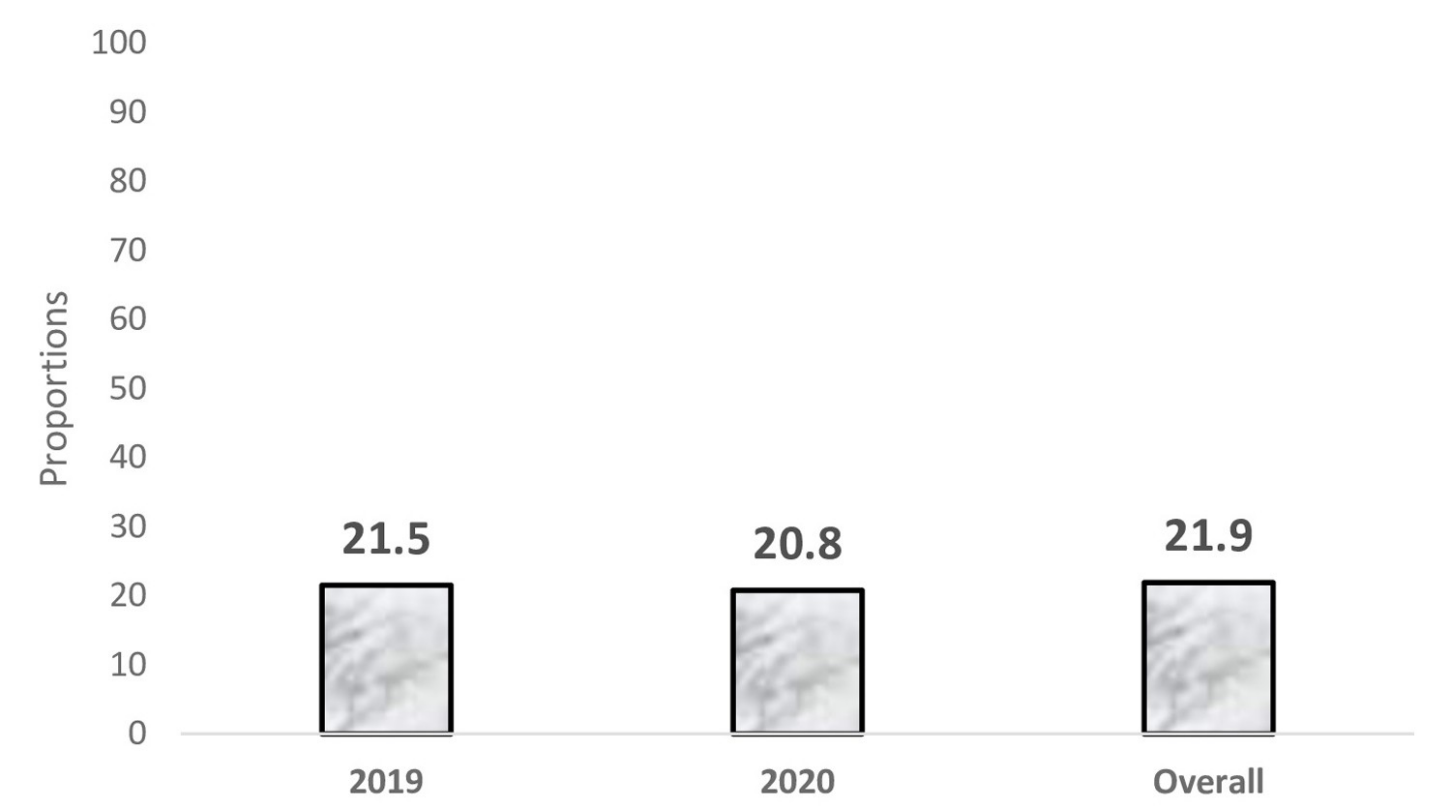
Proportion of HIV status among PWID (2019–2020).

The HIV status of participants in 2019, 2020 and overall proportions is shown in Fig 1 (Fig 1). This study included 2697 PWID, among which 571 participants (21.19%) tested positive for HIV. The HIV status among PWID in 2019 was 21.5% and 20.8% in 2020. The prevalence of HIV among participants who were married was 22.56%, among separated/divorced/ widowed was 34.85%, those who shared needles was 24.41% and those who did not use condom with regular sexual partner was 24.35%. The majority (38.6%) of HIV positive cases were among female participants and 19.5% among male participants.

### 4.2. Characteristics of study participants

The sociodemographic characteristics of the study participants are shown in Table 1. Most of the study participants were males (91.2%), and almost half (46.58%) of the participants were between the ages of 24–34 years. More than half of those who took part in the study were unmarried (59%). Fifty-four percent of participants had completed higher education (10–12 years), and about half of the participants (50.07%) were unemployed. A third (31.12%) of the participants reported having an average monthly income of less than INR 3000 and 26.85% reported having no income.

**Table 1:** Sociodemographic characteristics of HIV-positive PWID (n = 2697).

Characteristics	Total n (%)	Prevalence of HIV-positive PWID (%)	OR (95% CI)	*p*-value
**Year of survey**				
2019	1426(52.9)	21.5	1	
2020	1271 (47.1)	20.8	0.96 (0.80–1.15)	0.646
**Gender (n = 2697)**				
Male	2460 (91.2)	19.5	1	
Female	237 (8.8)	38.6	2.59 (1.95–3.43)	<0.001
**Age**				
18–24	959 (36.8)	17.4	1	
25–34	1213 (46.6)	21.5	1.44 (1.15–1.80)	<0.001
>35	432 (16.6)	27.6	1.80 (1.38–2.36)	<0.001
**Marital status (n = 2604)**				
Never married	1590 (59.0)	16.8	1	
Married	666 (24.7)	22.6	1.44 (1.15–1.80)	0.001
Separated/divorced/widowed	439 (16.3)	34.9	2.65 (2.09–3.35)	<0.001
**Education Status**				
Primary (0–6 years)	233 (8.6)	24.0	1	
Middle (7–9 years)	865 (32.1)	19.9	0.78 (0.55–1.10)	0.166
Higher (10–12 years)	1444 (53.6)	21.2	0.85 (0.61–1.17)	0.321
Graduate and above	152 (5.6)	23.7	0.98 (0.61–1.58)	0.937
**Employment status**				
Unemployed	1350 (50.1)	19.1	1	
Employed	777 (28.8)	25.7	1.47 (1.19–1.81)	<0.001
Self employed	569 (21.1)	19.9	1.05 (0.82–1.34)	0.687
**Average monthly income (INR) (n = 2670)**				
None	717 (26.8)	17.9	1	
<3000	831 (31.1)	24.4	1.48 (1.15–1.89)	0.002
3001–6000	662 (24.8)	17.8	0.99 (0.75–1.31)	0.97
6001–10,000	275 (10.3)	25.8	1.59 (1.14–2.22)	0.006
>10,000	185 (6.9)	21.1	1.22 (0.82–1.83)	0.322

Note: n = 2697, otherwise in parenthesis

### 4.3. Substance use and injecting behaviour

The prevalence and unadjusted odds ratio of substance use and injecting behaviours among HIV positive PWID are shown in Table 2. Heroin is the drug of choice among 98.92% of the participants. Among those who inject heroin 21.28% were HIV positive. More than half of the participants (54.80%) reported having shared needle/syringe and among those who shared needle/syringe, 24.42% had HIV infection. Concomitant alcohol use was high among 80.3% of the participants, and a significantly lower proportion of participants had HIV infection 19.2%. Needle/syringe sharing (*p*<0.001) and concomitant alcohol use (*p*<0.001) were significantly associated with HIV infection.

**Table 2:** Substance use and injecting behaviours among HIV positive PWID (n = 2697).

Characteristics	Total n(%)	Prevalence of HIV-positive PWID (%)	OR (95% CI)	*p*-value
**Injected any drugs in the past 3 months**				
No	159 (5.9)	32 (20.1)	1	
Yes	2535 (94.1)	539 (21.9)	1.07 (0.72–1.56)	0.730
**Type of drugs used (n = 2698)**				
Heroin	2662 (98.9)	568 (21.3)	1	
Parvon	29 (1.1)	2 (6.9)	0.27 (0.25–0.29)	0.077
**Sharing of needle/syringe (n = 2646)**				
No	1196 (45.2)	213 (17.8)	1	
Yes	1450 (54.8)	345 (24.4)	1.48 (1.22–1.79)	<0.001
**Concomitant alcohol use**				
No	531 (19.7)	154 (29.0)	1	
Yes	2164 (80.3)	415 (19.2)	0.58 (0.46–0.72)	<0.001

Note: n = 2697, otherwise in parenthesis

### 4.4. Sexual behaviours

Table 3 shows the results of the prevalence and unadjusted odds ratio of sexual behaviours among HIV positive PWID. According to this table, 16.7% of HIV positive participants reported having engaged in sex work. Of the 56.5% participants who reported condom use with regular sexual partner, 17.8% tested positive for HIV. Of those who did not use condom with regular partner, 24.4% had HIV infection. Of those who said they did not use a condom with regular paid partner, 27.2% had tested positive for HIV. Of the 59.0% participants who reported condom use with non-regular/ paid partner; 16.2% had HIV infection. Condom use with regular partner (p<0.001) and condom use with non-regular/paid partner (p<0.001) were significantly associated with HIV infection among PWID.

**Table 3:** Sexual behaviours among HIV positive PWID (n = 2697).

Characteristics	Total n (%)	Prevalence of HIV-positive PWID (%)	OR (95% CI)	*p*-value
**Whether engaged in sex work**				
No	2651 (98.4)	561 (21.9)	1	
Yes	42 (1.6)	7 (16.7)	0.74 (0.47–0.32)	0.479
**Condom used with regular partner (n = 2488)**				
No	1082 (43.5)	263 (24.4)	1	
Yes	1406 (56.5)	250 (17.8)	0.67 (0.55–0.82)	<0.001
**Condom used with non-regular/paid partner (n = 2495)**				
No	1022 (40.9)	277 (27.2)	1	
Yes	1473 (59.0)	239 (16.2)	0.52 (0.43–0.63)	<0.001

Note: n = 2697, otherwise in parenthesis

### 4.5. Factors associated with HIV infection among PWID

Table 4 shows the adjusted prevalence ratio and adjusted odds ratio of the factors associated with HIV infection among PWID. Only factors identified as significant were included in this analysis. HIV infection remained positively associated with being female (AOR = 1.74, 95% CI 1.26–2.41). We also found that being 35 years or older (AOR = 1.45, 95% CI 1.06–1.99), being married (AOR = 1.41, 95% CI 1.08–1.83) and being divorced/separated/widowed (AOR = 2.12, 95% CI 1.59–2.82) were associated with increased odds of HIV infection. Sharing needle/syringe remained positively associated with HIV infection (AOR = 1.62, 95% CI 1.30–2.00). Concomitant alcohol use was low among HIV positive PWID (AOR = 0.65, 95% CI 0.51–0.82), and a higher proportion of HIV positive PWID use condom with regular partner (AOR = 0.54, 95% CI 0.44–0.67). Adjusted prevalence ratio was also estimated from adjusted odds ratio. Hosmer-Lemeshow Goodness of Fit (GoF) statistic was p = 0.8467 which indicated a good logistic regression model fit. To justify for secular changes for 2019 and 2020 survey, we conducted a separate statistical analysis for the 2019 and 2020 survey and our result indicated that significant variables were similar in both surveys (S1 Table).

**Table 4:** Adjusted prevalence and odds ratio of the factors associated with HIV infection among HIV positive PWID.

Characteristics	Adjusted Prevalence ratio (95%CI)	AOR (95% CI)*			*p*-value
**Year of survey**					
2019	20.4 (18.1–22.8)	1			
2020	17.8 (15.5–20.1)	0.84 (0.68–1.04)			0.119
**Gender**					
Male	18.3 (16.7–20.0)	1			
Female	28.0 (21.7–34.2)	1.72(1.24–2.38)			0.001
**Age**					
18–24 years	16.7 (14.0–19.4)	1			
25–34 years	19.7 (17.3–22.2)	1.22 (0.95–1.57)			0.106
35+ years	23.2 (18.9–27.6)	1.50 (1.09–2.07)			0.012
**Marital Status**					
Never married	16.1 (14.1–18.2)	1			
Married	21.5 (18.0–25.1)	1.43 (1.09–1.85)			0.007
Divorced/separated	28.9 (23.9–33.7)	2.11 (1.58–2.81)			<0.001
**Sharing of needle/syringe**					
No	15.2 (13.0–17.5)	1			
Yes	22.7 (20.4–25.1)	1.63 (1.32–2.03)			<0.001
**Concomitant alcohol use**					
No	24.8 (20.9–28.7)	1			
Yes	17.8 (16.0–19.7)	0.65 (0.51–0.82)			<0.001
**Condom use with regular partner**					
No	25.3 (22.4–28.2)	1			
Yes	15.6 (13.6–17.5)	0.54 (0.44–0.67)			<0.001

*Confounding factors adjusted are: education, employment, average monthly income, type of drugs used, injected drugs in past 3 months, engaged in sex work and condom use with non-regular/paid partner.

## 5. Discussion

Evidence to help support the targeting of intervention to reduce HIV among drug users in Mizoram is limited. This study found that over 1 in 5 PWID who resided in Mizoram had HIV and that being female, over the age of 35, married or previously married and sharing needle and syringe reported higher odds of HIV among PWID.

The prevalence of HIV among PWID reported in this study was twice those reported in the latest Integrated Biological and Behavioural Surveillance (IBBS) among PWID in Mizoram [35]. However, there is no recent published data on the prevalence of HIV among PWID in Mizoram to compare with our study findings, but a recent nationwide HIV estimates report revealed that the HIV prevalence and incidence among adults in Mizoram were the highest in India [23]. The increased prevalence of HIV among the PWID population could be multi-factorial such as; decreased AIDS-related mortality rate [23], discrimination, stigma and marginalization [36]. Criminalization and stigmatization among PWID contribute to HIV epidemics as this may linked to difficulty accessing harm reduction and other public health services [16, 37]. Similar to our findings, recent studies conducted in Scotland [38] and Romania [39] have also reported an increase in HIV among PWID. The common attributes reported by these two studies as the possible cause of increase in HIV prevalence among PWID included low or difficult access to harm reduction services, often due to chaotic lifestyles [38].

We also found the odds of HIV among PWID were lower in 2020 compared with 2019, but this reduction did not differ statistically. The possible explanation for the low prevalence in 2020 could be attributed to the current COVID-19 pandemic and its subsequent lockdown; which had negative impact on the well-being, mental health and social support on individuals [40] including PWID from gathering in hotspot areas and disrupted the collection of data. Poor access to services during COVID-19 pandemic when services were closed or limited for some periods of time could also be another reason for needle/syringe sharing. However, the exact effect of the pandemic on HIV among PWID is unknown, and further studies need to look into this.

We found that female PWID were two times more likely to report HIV infection than male PWID. This finding is similar to other cross-sectional studies done in Tanzania and South Africa showing that women are more vulnerable to HIV infection [30, 41, 42]. The vulnerability of females who inject drugs to HIV infection has been recognized to be due to multiple layers of risks such as unsafe sexual practices, multiple sexual partners, engaging in commercial sex work to be able to purchase drugs [30], and intimate partner violence including physical assaults, sexual coercion and rape from their partner [43, 44].

The findings of our study also suggested that being over the age of 35 was a factor associated with higher reported HIV among PWID. This finding is consistent with other similar cross-sectional studies done in India [45] and Cambodia [46]. This may be attributed to the fact that older PWID may have spent many years injecting drugs and have had longer exposure to drugs and risky behaviours for infection [47].

Our study also found that divorced/separated/widowed PWID had twice the odds of HIV infection compared to never married PWID, which is similar to the study done in South Africa [48]. A plausible explanation is that divorced/separated/widowed PWID have a wider sexual network, leading to more sexual partners which in turn increases their risk of HIV/AIDS [49]. The term sexual network describes a group of individuals connected through sexual contact [50]. It was also interesting to find that PWID who were married had higher odds of HIV infection than those who were not married. This is similar to a study done in South Africa that found HIV prevalence among a significant proportion of married people [51]. Clearly, the risk of HIV in married people is directly related to sexual practices, however, understanding the socio-cultural context is important in explaining this. The relationship between married people and HIV infection is complex and requires further study.

This study showed that a large proportion of PWID still engaged in sharing needles/syringes despite the efforts made by MSACS in promoting harm reduction services, including needle syringe exchange program. This is particularly concerning given that all the participant in this analysis are living with HIV. There appears to be a confluence of factors that renders PWIDs to participate in unsafe injecting behaviours, such as; lack of access to new needles/syringes, inconsistent supply of needles/syringes from intervention projects and participating NGOs [52, 53], criminalization and harassment from anti-drug groups, poverty and unemployment [52, 54].

Our study found that concomitant alcohol use was low among HIV positive PWID. This is in contrast to a study done in Vietnam [55] which demonstrated that alcohol use was highly prevalent among HIV-infected men who inject drugs. The impact of alcohol use on HIV outcomes is uncertain, however, evidence suggests that there are possible mechanisms by which alcohol may be related to HIV disease progressions, such as low medication adherence and suboptimal retention in care [56–58]. Those who use alcohol may be less likely to adhere to antiretroviral treatment (ART) and also may be less able to tolerate ART due to hepatotoxicity [57, 59]. Majority of HIV positive PWID in this study may be on ART and hence alcohol use was low among them.

This study also found that a higher proportion of HIV positive PWID uses condom with their regular partner. This finding is similar to other studies done in India [53, 60] which reported a reduction in risk behaviours including having unprotected sex among PWID who tested positive for HIV after collecting their test. This suggests that sharing contaminated injecting equipment is a more important determinant of HIV infection among PWID. However, IBBS reported condom use with both casual and regular partner was low, with only 15% having used condoms consistently with their regular partner [35]. This highlights that condom programmes targeted at PWIDs are nevertheless essential to prevent transmission of HIV to their sexual partners.

The strength of this study lies in the fact that this is the first study that looks at prevalence and factors associated with HIV among HIV positive PWID in Mizoram, northeast India. Secondly, this study used the most recent data from MSACS. Thirdly, the study can be generalizable to the PWID population in Mizoram due to large sample size collected across 8 districts in Mizoram. However, there are also some limitations to this study. Firstly, the study’s cross-sectional nature provides a snapshot of the prevalence of HIV infection among PWID and does not allow for the establishment of temporality or causality between HIV infection and the predictors of interest. Secondly, desirability bias is possible, with respondents giving socially desirable responses rather than the truth. Thirdly, there is a potential for selection bias as participants who were only willing to participate in TI services were included in this study. Fourthly, this study is generalizable only to PWID population in Mizoram, comparison with other states in India were not made. Lastly, because these data were cross-sectional, we did not have data on the number of injection partners and years of injection.

## 6. Policy implications

The findings of this study suggest that sharing injecting equipment is a more important determinant of HIV infection among PWID in Mizoram. The role of harm reduction services such as needle/syringe exchange program, opioid substitution therapy and ART are well established [61]. An assessment of the existing implementation mechanism to understand the insufficient and inconsistent provision of services/commodities is required. Focused attention and increased funding for the provision of clean needle/syringe services and easy accessibility of these services among PWID is necessary [53]. Education and risk reduction counselling on avoiding needle/syringe sharing and the importance of using clean injecting equipment needs to be emphasized among HIV positive PWID [52]. The prevention of HIV among PWID cannot be achieved through one program or service alone but requires a combination of interventions [62].

The combination of injecting drug behaviour and unsafe sexual practices among female PWID has made them more vulnerable to HIV infection, underscoring the need for tailored delivery of services and harm reduction packages among women [63]. Strategies geared specifically for women that include partner counselling, safe sex education, domestic violence and rape prevention, drug treatment reduction strategies and centers that cater specifically for women should be emphasized and integrated with other harm reduction strategies [64]. With the growing number of women who inject drugs in Mizoram, strategies to reach and engage women in HIV prevention interventions are needed urgently.

## 7. Conclusion

This study suggests that there is a high prevalence of HIV among PWID in Mizoram. HIV among PWID was significantly higher among female, older participants and divorced/separated/widowed participants. A large proportion of PWID in this study still engages in sharing needles/syringes. We suggest that to prevent and control HIV infection among PWID in Mizoram, harm reduction services and interventions should be tailored to emphasize strategies to prevent needle/sharing and provide an uninterrupted supply of clean needle/syringe [53]. Interventions to reduce HIV among PWID should also target females and provide harm reduction packages geared specifically for female PWID [64].

## Supporting information

S1 Table 2019 and 2020 surveys of the factors associated with HIV among HIV positive PWID. (DOCX)

S1 Dataset (XLSX)
