# Supplementary material for: HIV and its associated factors among people who inject drugs in Mizoram, Northeast India
Source: PLoS One. 2023 May 22;18(5):e0286009. doi: 10.1371/journal.pone.0286009 (PMC10202280; doi:10.1371/journal.pone.0286009)
Supplement: S1 Table — (DOCX) [file pone.0286009.s001.docx]

| S1 Table: 2019 and 2020 surveys of the factors associated with HIV among HIV positive PWID | | | | |  |
| --- | --- | --- | --- | --- | --- |
|  |  |  |  |  |  |
| Characteristics | 2019 | | 2020 | |  |
|  | OR (95% CI) | p-value | OR (95% CI) | p-value |  |
| **Gender** |  |  |  |  |  |
| Male | 1 |  | 1 |  |  |
| Female | 1.95 (1.30-2.93) | 0.001 | 1.58 (0.94-2.65) | 0.082 |  |
| **Age** |  |  |  |  |  |
| 18-24 | 1 |  | 1 |  |  |
| 25-34 | 1.16 (0.86-1.57) | 0.319 | 1.27 (0.84-1.90) | 0.245 |  |
| >35 | 1.32 (0.95-1.98) | 0.089 | 1.33 (0.83-2.12) | 0.232 |  |
| **Marital status** |  |  |  |  |  |
| Never married | 1 |  | 1 |  |  |
| Married | 0.95 (0.66-1.37) | 0.797 | 2.20 (1.51-3.21) | <0.001 |  |
| Separated/divorced/widowed | 1.37 (0.95-1.98) | 0.089 | 4.23 (2.71-6.62) | <0.001 |  |
| **Sharing of needle/syringe** |  |  |  |  |  |
| No | 1 |  | 1 |  |  |
| Yes | 1.53 (1.17-2.01) | 0.002 | 1.44 (1.04-2.01) | 0.028 |  |
| **Concomitant alcohol use** |  |  |  |  |  |
| No | 1 |  | 1 |  |  |
| Yes | 0.71 (0.52-0.97) | 0.03 | 0.65 (0.44-0.95) | 0.028 |  |
| **Condom use with regular partner** |  |  |  |  |  |
| No | - |  | 1 |  |  |
| Yes | - |  | 0.24 (0.17-0.35) | <0.001 |  |
